# A novel *MC1R* allele for black coat colour reveals the Polynesian ancestry and hybridization patterns of Hawaiian feral pigs

**DOI:** 10.1098/rsos.160304

**Published:** 2016-09-07

**Authors:** Anna Linderholm, Daisy Spencer, Vincent Battista, Laurent Frantz, Ross Barnett, Robert C. Fleischer, Helen F. James, Dave Duffy, Jed P. Sparks, David R. Clements, Leif Andersson, Keith Dobney, Jennifer A. Leonard, Greger Larson

**Affiliations:** 1 The Palaeogenomics and Bio-Archaeology Research Network, Research Laboratory for Archaeology, University of Oxford, Dyson Perrins Building, South Parks Road, Oxford OX1 3QY, UK; 2 Center for Conservation Genomics, Smithsonian Conservation Biology Institute, National Zoological Park, MRC 5508, Washington, DC 20013-7012, USA; 3 Department of Vertebrate Zoology, National Museum of Natural History, Smithsonian Institution, PO Box 37012, Washington, DC 20013-7012, USA; 4 Botany Department, University of Hawaìi at Manoa, 3190 Maile Way, Room 101, Honolulu, HI 96822, USA; 5 Department of Biology, Trinity Western University, Langley, BC, CanadaV2Y 1Y1; 6 Department of Medical Biochemistry and Microbiology, Uppsala University, 75123 Uppsala, Sweden; 7 Department of Archaeology, Classics and Egyptology, University of Liverpool, 12–14 Abercromby Square, Liverpool L69 7WZ, UK; 8 Conservation and Evolutionary Genetics Group, Estación Biológica de Doñana (EBD-CSIC), Avenida Américo Vespucio, s/n, 41092 Seville, Spain; 9 Department of Ecology and Evolution, Cornell University, Ithaca, NY, USA; 10 Discipline of Archaeology, National University of Ireland, University Road, Galway, Ireland; 11 The Bioarchaeology and Genomics Laboratory, Department of Anthropology, Texas A&M University, MS 4352 TAMU, College Station, TX 77843-4352, USA

**Keywords:** *Sus scrofa*, mitochondrial DNA, Hawaii, feral pigs, Pacific colonization

## Abstract

Pigs (*Sus scrofa*) have played an important cultural role in Hawaii since Polynesians first introduced them in approximately AD 1200. Additional varieties of pigs were introduced following Captain Cook's arrival in Hawaii in 1778 and it has been suggested that the current pig population may descend primarily, or even exclusively, from European pigs. Although populations of feral pigs today are an important source of recreational hunting on all of the major islands, they also negatively impact native plants and animals. As a result, understanding the origins of these feral pig populations has significant ramifications for discussions concerning conservation management, identity and cultural continuity on the islands. Here, we analysed a neutral mitochondrial marker and a functional nuclear coat colour marker in 57 feral Hawaiian pigs. Through the identification of a new mutation in the *MC1R* gene that results in black coloration, we demonstrate that Hawaiian feral pigs are mostly the descendants of those originally introduced during Polynesian settlement, though there is evidence for some admixture. As such, extant Hawaiian pigs represent a unique historical lineage that is not exclusively descended from feral pigs of European origin.

## 1. Introduction

The global colonization process that began when modern humans first left Africa eventually led to the establishment of human settlements across the Pacific [1]. Domestic and commensal animals accompanied Polynesian (and later Eurasian) voyagers into the Pacific over the last two and a half millennia. Though these organisms facilitated the survival of the human colonists both during their voyage and after they arrived, domestic populations also had considerable (often destructive) ecological consequences as invasive species on the islands onto which they were introduced [2–4].

A recent examination of the radiocarbon dating evidence suggests that the Hawaiian islands were first colonized by Polynesians less than 800 years ago [1]. The domestic pigs [5] (*Sus scrofa*), dogs [6] (*Canis familiaris*) and chickens, e.g. [6] (*Gallus gallus*) that they introduced all must have derived from East Asian progenitors, and some pigs established feral populations. Captain Cook arrived in 1778 [7] and introduced larger and potentially more environmentally destructive European domestic pigs to the islands that some authors have speculated replaced the original Polynesian pig [8]. Few studies, however, have attempted to test this hypothesis or quantify the degree to which the more recent European pigs introgressed into local Polynesian pig populations [9].

Modern Hawaiian pig populations survive primarily as feral groups that cause extensive ecological damage to the native Hawaiian ecosystem [10,11], especially in forested environments [3,12,13]. These pigs alter native ecosystems [14] by damaging and consuming endemic plant species and by altering soil fertility [15]. They assist in the dispersal of invasive plants, prey on the eggs of native ground nesting birds and create microhabitats that allow invasive mosquitoes carrying avian malaria to breed [16].

Human attitudes towards pigs in Hawaii have changed dramatically over the past century. Hawaiian feral pigs retain significant cultural importance linked directly to their long history as iconic Polynesian feast animals dating back to the first human colonization of the archipelago [17]. The recognition of their harmful ecological role, however, and the subsequent management practices put in place to mitigate those effects have led to a more recent polarization of opinion [2,13]. As a result, arguments regarding the ancestry of these pigs have arisen as part of the discussion about how to manage them in a way that is both culturally sensitive, and that allows for the survival of native Hawaiian fauna and flora.

While it has been argued that the feral pigs on the islands do not represent ‘real’ Polynesian pigs and thus should be eradicated [8], others contend that the pigs are culturally important, and should be protected [18]. In addition, debates regarding animal cruelty and the value of pigs to hunting advocates have further increased tensions [2]. Understanding the degree to which modern feral pigs retain their Polynesian ancestry and whether pigs introduced by Europeans have replaced those originally introduced will lead to more informed debates regarding the management of Hawaiian pigs.

Here, in order to test the hypothesis that the majority of extant Hawaiian feral pigs are descended primarily from European stock, we sequenced and analysed both the mtDNA control region and the *melanocortin 1 receptor (MC1R)* gene in 57 feral pigs sampled from four Hawaiian Islands (Kauai, Oahu, Molokai and Hawaii). The *MC1R* gene, a transmembrane G-protein coupled receptor expressed primarily in melanocytes and melanoma cells, underlies feather, skin and coat colours by determining black/red pigment switching [19]. A previous study of *MC1R* variation in pigs revealed 13 alleles in wild and domestic populations in Europe and Asia [20], and this gene has also been used to detect hybrids between wild and domestic populations, e.g. [21]. We first reconstructed a phylogeny utilizing the mtDNA control region data generated here (alongside a diverse set of publically available mtDNA sequences). We then reconstructed a network of novel and previously sequenced *MC1R* alleles. Using these data, we then determined the relative contributions of pigs introduced by Polynesians and Europeans to the modern gene pool.

## 2. Material and methods

### 2.1. Material

Tissue samples for DNA analysis were collected from 57 feral pigs legally hunted in 13 locations across the Hawaiian archipelago (electronic supplementary material, table S1). These samples represent four of the six Hawaiian Islands with extant feral pig populations: Niihau, Kauai, Oahu, Molokai, Maui and Hawaii [22]. Phenotypic information, including sex, age and coat colour, was recorded by each hunter and is listed for each of the 47 samples from which DNA was successfully sequenced (electronic supplementary material, table S1).

### 2.2. Extraction

DNA was extracted from 200 mg of muscle tissue (cut into small pieces). First, the tissue sample was digested in 300 µl of extraction buffer (0.1 M NaCl, 10 mM Tris-HCl pH 8.0, 0.5% SDS) and 0.06 mg of proteinase K rotating at 50°C overnight. The DNA was then extracted by salting out: 80 µl of saturated NaCl was added and the mix was vortexed and spun at 9000 r.p.m for 10 min. The supernatant was transferred to a new tube and the step was repeated one or two times. The extract was then ethanol precipitated and re-suspended in 200 µl of 1× TE buffer.

### 2.3. Amplification

A 700 bp fragment of the 5′ end of the mtDNA control region was amplified using two overlapping fragments generated using primer combinations L15387/H764 (5′-CTCCGCCATCAGCACCCAAAG-3′/5′-TGCTGGTTTCACGCGGCA-3′) and L119n/H16108n (5′-CAGTCAACATGCATATCACC-3′/5′-GCACCTTGTTTGGATTRTCG-3′) [23]. In addition, a 1000 bp fragment of *MC1R* was amplified using the two primers Epig16/PCR2 (5′-GGGAAGCTTGACCCCCGAGAGCGACGCGCC-3′/5′-CGCCGTCTCTCCAGCCTCCCCCACTC-3′) [24]. PCR amplifications were performed in 25 µl reactions containing 1 × PCR Gold Buffer (Applied Biosystems), 2.5 mM MgCl_2_, 1 M Betain (2.5 mg ml^−1^) (stock concentration, Fisher Scientific), 0.625 mM dNTP, 0.5 µM of each primer, 1.25 U AmpliTaq Gold (Applied Biosystems) and 1 µl of DNA extract.

PCR thermal cycling conditions for the mitochondrial primer pairs consisted of a 90 s denaturation step at 94°C, followed by 45 cycles of 45 s denaturation at 95°C, 45 s annealing at 56°C, 45 s extension at 72°C and then a final 5 min extension step at 72°C. The same conditions were used for the *MC1R* primers except that the annealing temperature was 63°C. No contamination was detected in any of the extraction or PCR controls. The sequences were generated by Sanger sequencing using the same primers used in the PCR reactions on an ABI 3730 capillary sequencer.

### 2.4. Phylogenetic reconstruction

**Figure 1.**
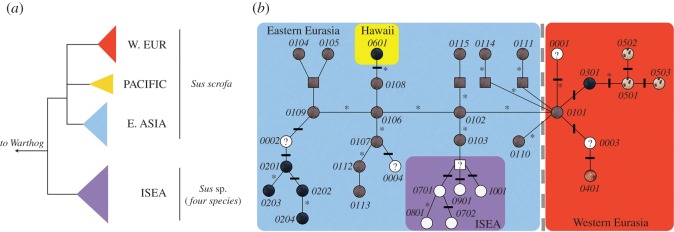
A phylogenetic tree of mitochondrial sequences (*a*) and a median-joining network (*b*) of global pig *MC1R* alleles. Eastern Eurasian, Island Southeast Asian (ISEA), Hawaiian and Western Eurasian alleles are outlined by blue, purple, yellow and red, respectively. Nomenclature follows [20], where the first two digits in the four number sequence represent coat colour, and the last two digits differentiate each allele such that *01xx* represents *Sus scrofa* wild-type coat: *02xx* represents Asian Dominant Black, *03xx* is European Dominant Black, *04xx* is Recessive Red, *05xx* is Spotted Coat and *06xx* is Hawaiian Dominant Black. Numbers 07 through 10 denote *Sus barbatus, S. verrucosus, S. celebensis* and *S. cebifrons*, respectively. Open nodes with question marks, denoted by *00xx*, represent nucleotide sequences for which there was no phenotypic information. Each branch between nodes represents a single synonymous change. Black ticks perpendicular to each branch represent a non-synonymous mutation that changes the *MC1R* protein sequence. Asterisks near each branch represent mutations that also occur elsewhere in the network. Additional detail is presented in electronic supplementary material, figures S1–S3.

The mtDNA sequences were aligned alongside 223 reference sequences (electronic supplementary material, table S2) corresponding to known European, Asian and Pacific mtDNA haplotypes in *Geneious* (Biomatters Ltd 2005–2011). Neighbour-Joining and Bayesian trees were constructed using *Geneious* and *MrBayes* [25], respectively. Bayesian trees were generated under a Tamura & Nei substitution model (best model inferred from ModelTest [26]). Parallel runs of five million MCMC samples (10% burnin) were drawn. Topology and posterior/bootstrap values for each node on the trees (visualized in FigTree v. 1.4.1 [27]) mirrored those generated in previous studies [5,28]. For simplicity, individual branches were collapsed to depict the relationships between clades of individuals from Western Europe, East Asia, the Pacific and other species of *Sus* (figure 1*a*).

The 38 *MC1R* sequences generated as part of this study were aligned alongside 13 reference alleles previously described in [20] as well as a total of 348 publically available sequences including *MC1R* sequences obtained from previously published *Sus* genomes [29,30] (electronic supplementary material, table S3; ENA accession: ERP001813). Extraction, filtering and consensus call from BAM files were made using Samtools [31] as previously described in [29]. The *MC1R* network (figure 1*b*) was constructed using a total of 400 sequences (electronic supplementary material, tables S3–S5) and synonymous and non-synonymous mutations for the 34 known *MC1R* alleles presented in electronic supplementary material, table S2 of [20], were cross-referenced with the network diagram.

## 3. Results

Of the 57 total Hawaiian samples, 47 yielded mtDNA control region sequences. Of those, 33 (70%) individuals possessed mitochondrial haplotypes belonging to a previously identified Pacific Clade [32]. These haplotypes were also present on all four islands included in this study. Four samples possessed European mitochondrial haplotypes and 10 possessed East Asian haplotypes (figure 1; electronic supplementary material, table S1).

*MC1R* sequences were generated from 38 of the 47 samples that produced mitochondrial data, and then aligned with 348 sequences derived from pigs of Eurasian and Southeast Asian origin (electronic supplementary material, table S3). Of the 38 samples, 15 possessed at least one variant found in European domestic pigs, and only four possessed exclusively European domestic alleles (even if the precise allele could not be determined) (electronic supplementary material, table S1) [20].

In addition, of the 38 individuals, 34 (23 of which were homozygotes and 11 of which were heterozygotes) possessed a previously undescribed allele that we designate 0601. Relative to the European wild-type haplotype (0101), this allele possessed three synonymous and one non-synonymous mutations. The synonymous mutations leading to alleles 0102, 0106 and 0108 alter the third base positions at codons 121, 17 and 207, respectively (figure 1*b*; electronic supplementary material, figures S1–S3).

The non-synonymous mutation, a guanine to adenosine shift occurs at the first base of codon 124. This identical mutation was previously shown to have occurred in European pigs (leading from allele 0101 to 0301) and alters codon 124 from Aspartic acid to Asparagine [20] resulting in black colouring in European domestic pigs (figure 1*b*). All the pigs for which colour was recorded (electronic supplementary material, table S1) and that possessed the novel allele and were black, suggesting that the mutation is associated with black colouring in both European domestic and Hawaiian feral pigs.

**Figure 2.**
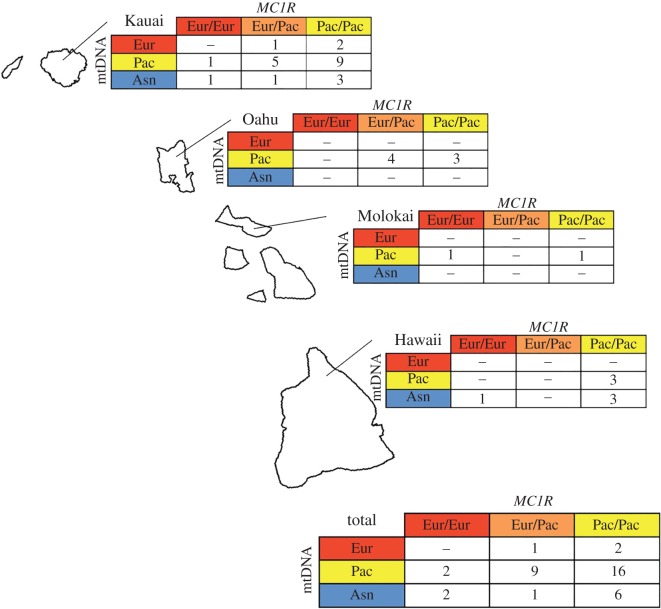
A map of Hawaii depicting the number of samples on each island and the combination of mtDNA and *MC1R* sequences possessed be each sample. Red, Yellow, and Blue generally represent Western Eurasian, Pacific, and East Asian origins respectively for each locus. The abbreviation Pac refers to the mitochondrial Pacific Clade and to the Hawaiian *MC1R* allele depicted in figure 1.

The data presented here suggest that this identical mutation most likely occurred independently on European and Asian *MC1R* haplotypes. The alternative possibility that the allele was generated by intragenic recombination between a European allele carrying the non-synonymous change at codon 124 and an Asian allele carrying the three synonymous changes is less likely given that the mutation occurs at a codon flanked on either side by Asian sequence. In addition, none of the pigs sampled on Hawaiian islands possessed an Asian *MC1R* allele that was not the newly identified 0601 suggesting a lack of Asian templates with which the European mutation could have recombined. Lastly, though the overall dataset is out of Hardy–Weinberg (HW) equilibrium, the alleles were not significantly out of HW on any individual island (figure 2).

## 4. Discussion

### 4.1. The molecular basis for black coats on Hawaii

A recent study investigating the allelic variation in the coat colour gene *MC1R* in wild boar and domestic pigs across Eurasia demonstrated that while all *MC1R* haplotypes found in wild boar populations across Eurasia are differentiated solely by synonymous mutations, virtually all of the mutations in domestic pigs are non-synonymous [20]. This pattern suggests that purifying selection in wild populations eliminates the non-synonymous mutations that, in a human context, are actively promoted [20,33].

In addition, domestic pigs with black coat colours are present in both Asia and Europe. The observation that recurrent mutations on separate *MC1R* allelic backgrounds lead to black coat colour provided supporting evidence for the independent domestication of geographically and genetically differentiated wild boar populations in Western and Eastern Eurasia. This conclusion has also been reached from studies using both mtDNA [23,34] and nuclear DNA [29,34].

The temporal and geographical origins (within East Asia) of the novel *MC1R* mutation described here, and its acquisition by the ancestors of Polynesian pigs, are unknown. Regardless, our results suggest that humans across the Old World independently selected for black coat coloration and that this was achieved by selecting for three separate haplotypes, each of which results in black coats. This human penchant for novelty often manifests itself though the selection for colour variants in both domestic plants and animals that natural selection actively eliminates [33].

### 4.2. Patterns of gene flow among pigs introduced to Hawaii

Phylogeographic studies employing mitochondrial markers in pigs have demonstrated a close association between geographical provenance and phylogenetic clade affinities. More specifically, numerous studies have revealed geographical clades corresponding to Western Eurasia and East Asia, and within East Asia, a well-supported Pacific Clade has also been identified (figure 1*a*) [5,23,28]. This clade includes modern and ancient pig samples found across Island Southeast Asia (ISEA) and the Pacific including Hawaii [32]. Most (70%) of the Hawaiian pigs sequenced as part of this study cluster in the Pacific Clade, and 30% belong to the Asian and Western Eurasian clades. The presence of European and East Asian sequences is probably the result of admixture between the pigs first brought by Polynesians, and those introduced in the last few hundred years (including the progeny of ‘improved breeds’) that derive their ancestry from Europe and Asia [35]. The predominance of the Pacific clade haplotypes, however, suggests that the original Polynesian lineages have not been completely replaced by more recent introductions.

Western explorers, like the Polynesians before them, travelled with and introduced domesticated plants and animals across the Pacific. In many cases, Westerners came into contact with local cultures that already possessed domesticated varieties of the same taxa that led to gene flow and possibly replacement. On Tahiti, pigs became notably larger only 3 years after Europeans introduced new stock in 1774 [36], and morphological studies carried out on Polynesian pigs concluded that the prehistoric Polynesian pig population had been mostly replaced by the more recently introduced Eurasian breeds [10,13,37].

The genetic evidence presented here indicates that the current Hawaiian feral pig population is a mixture of those brought to Hawaii by the Polynesians and pigs of European (and possibly Asian) origin introduced to the islands much later. Our data demonstrate that of the 38 feral pigs from which we sequenced both mitochondrial and *MC1R* sequences, only two possess markers derived exclusively from a non-Pacific source. As such, the vast majority of the feral pigs on Hawaii represent a globally rare domestic lineage.

Ethnographic, historical and archaeological evidence suggests that the first introduced pigs were husbanded animals, and not hunted ones. And though the issue of whether the first pigs on Hawaii became feral prior to the arrival of Europeans remains contentious, extensive damage to native habitats by feral pigs appears to be recent [38]. In fact, it was probably not until the twentieth century, with the introduction of new sources of protein such as earthworms and invasive fleshy-fruited plants that pigs were able to thrive in the forests, thus becoming a significant problem to the native flora and fauna [38].

Other rare pig lineages including the Lanyu pig [39] (a type of pig supposedly indigenous to the island of Lanyu off the coast of Taiwan) are now the subject of specific conservation and management plans within an economic, cultural and scientific framework. Hawaiian pigs are clearly unique with respect to their origins and genetic characteristics, a fact that will have ramifications for determining the management strategies associated with their continued existence on Hawaii.

## Supplementary Material

Figure S1

## Supplementary Material

Figure S2

## Supplementary Material

Figure S3

## Supplementary Material

Supp Figure legends

## Supplementary Material

Table S1

## Supplementary Material

Table S2

## Supplementary Material

Table S3

## Supplementary Material

Table S4

## Supplementary Material

Table S5

## Data accessibility

The mtDNA and *MC1R* sequences generated as part of this project are available on GenBank, accessions KX674380-KX674464.
