# Supplementary figures and images for: A novel MC1R allele for black coat colour reveals the Polynesian ancestry and hybridization patterns of Hawaiian feral pigs
Source: R Soc Open Sci. 2016 Sep 7;3(9):160304. doi: 10.1098/rsos.160304 (PMC5043315; doi:10.1098/rsos.160304)

# EASTERN EURASIA

# HAWAII

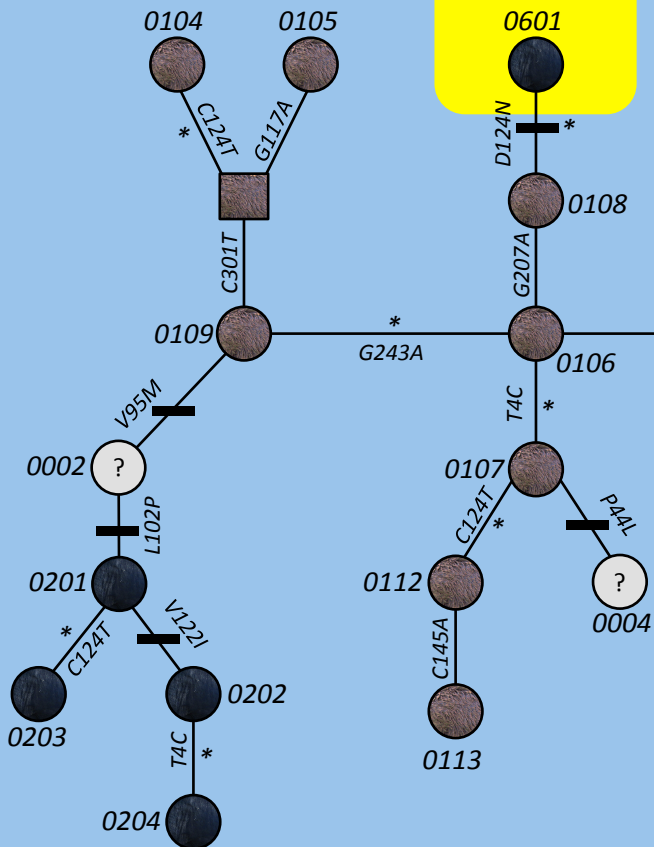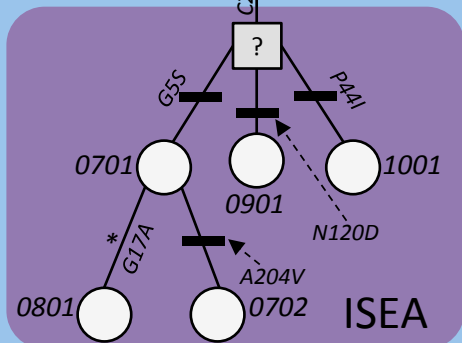

# ISEA

# WESTERN EURASIA

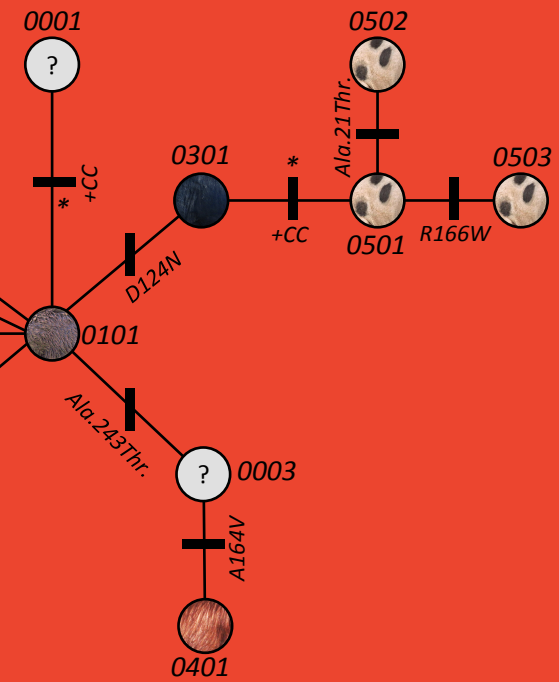

Supplement: Figure S1 [file rsos160304supp1.pdf]

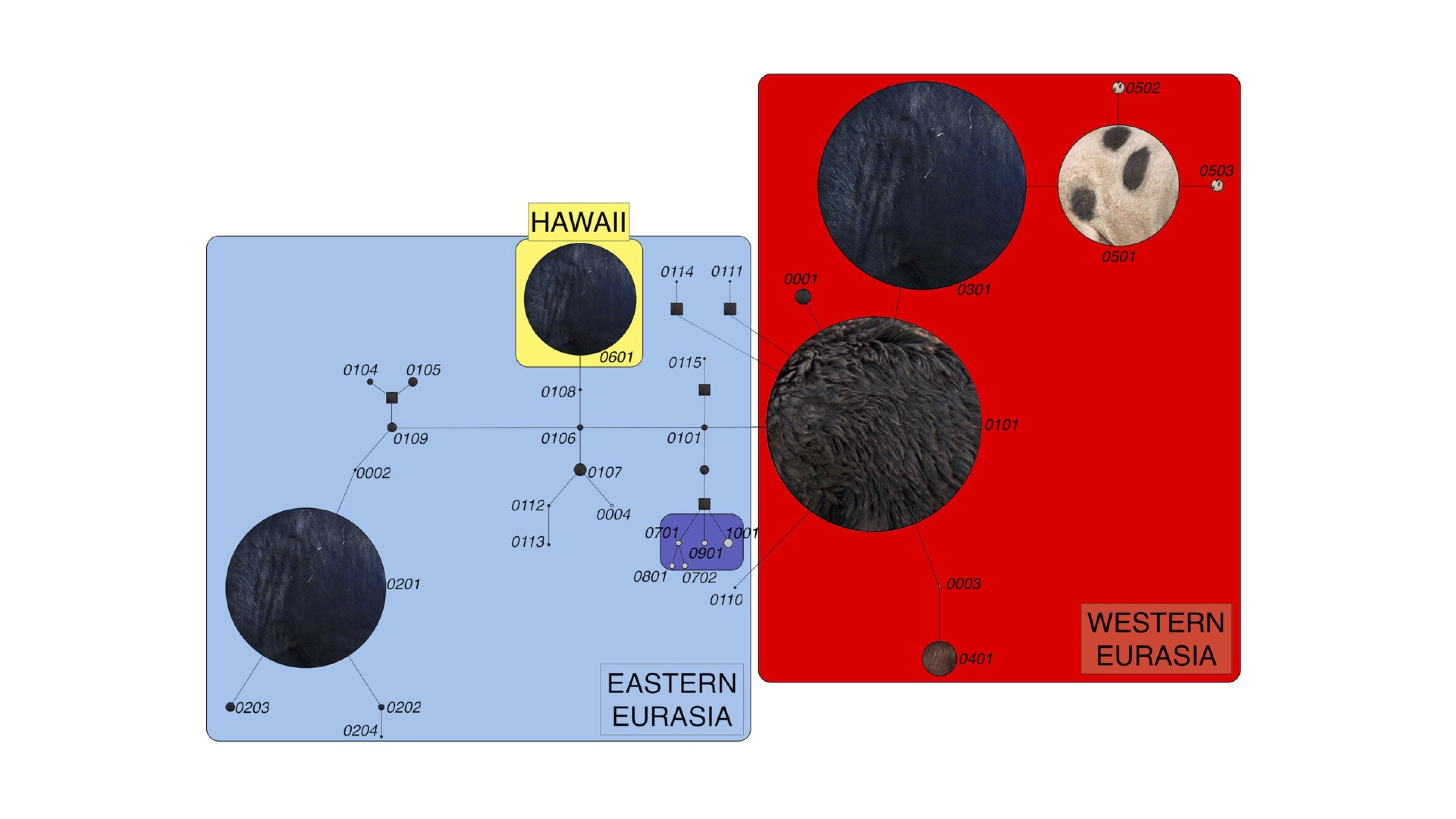

Supplement: Figure S3 [file rsos160304supp3.jpg]
