# Supplementary material for: A novel MC1R allele for black coat colour reveals the Polynesian ancestry and hybridization patterns of Hawaiian feral pigs
Source: R Soc Open Sci. 2016 Sep 7;3(9):160304. doi: 10.1098/rsos.160304 (PMC5043315; doi:10.1098/rsos.160304)
Supplement: Figure S2 [file rsos160304supp2.pdf]

Supp. Table 2

| | Phenotypes | MC1R Allele | Codon Number and Coding Sequence | | | | | | | | | | | | | | | | | | | | | | | |
|---|---|---|---|---|---|---|---|---|---|---|---|---|---|---|---|---|---|---|---|---|---|---|---|---|---|---|
| | | | 2 | 4 | 5 | 17 | 20 | 21 | 22 | 44 | 64 | 80 | 95 | 102 | 117 | 121 | 122 | 124 | 145 | 164 | 166 | 168 | 204 | 207 | 243 | 301 |
| *Sus scrofa* | *Wild type* | *0101/E+* | CCT | CTT | GCC | GCG | GCC | GCC | CCC | CCC | GCC | GTC | GTG | CTG | CAG | AAT | GTC | GAC | CGC | GCG | CGG | ATC | GCG | GCG | GCG | TAC |
| | | | *Pro* | *Leu* | *Gly* | *Ala* | *Ala* | *Ala* | *Pro* | *Pro* | *Ala* | *Val* | *Val* | *Leu* | *Gln* | *Asn* | *Val* | *Asp* | *Arg* | *Ala* | *Arg* | *Ile* | *Ala* | *Ala* | *Ala* | *Tyr* |
| | | *0102/E+* | ... | ... | ... | ... | ... | ... | ... | ... | ... | ... | ... | ... | ... | ..C | ... | ... | ... | ... | ... | ... | ... | ... | ... | ... |
| | | | " | " | " | " | " | " | " | " | " | " | " | " | " | " | " | " | " | " | " | " | " | " | " | " |
| | | *0103/E+* | ... | ..C | ... | ... | ... | ... | ... | ... | ... | ... | ... | ... | ... | ..C | ... | ... | ... | ... | ... | ... | ... | ... | ... | ... |
| | | | " | " | " | " | " | " | " | " | " | " | " | " | " | " | " | " | " | " | " | " | " | " | " | " |
| | | *0104/E+* | ... | ... | ... | ..A | ... | ... | ... | ... | ... | ... | ... | ... | ... | ..C | ... | ..T | ... | ... | ... | ... | ... | ... | ..A | ..T |
| | | | " | " | " | " | " | " | " | " | " | " | " | " | " | " | " | " | " | " | " | " | " | " | " | " |
| | | *0105/E+* | ... | ... | ... | ..A | ... | ... | ... | ... | ... | ... | ... | ... | ..A | ..C | ... | ... | ... | ... | ... | ... | ... | ... | ..A | ..T |
| | | | " | " | " | " | " | " | " | " | " | " | " | " | " | " | " | " | " | " | " | " | " | " | " | " |
| | | *0106/E+* | ... | ... | ... | ..A | ... | ... | ... | ... | ... | ... | ... | ... | ... | ..C | ... | ... | ... | ... | ... | ... | ... | ... | ... | ... |
| | | | " | " | " | " | " | " | " | " | " | " | " | " | " | " | " | " | " | " | " | " | " | " | " | " |
| | | *0107/E+* | ... | ..C | ... | ..A | ... | ... | ... | ... | ... | ... | ... | ... | ... | ..C | ... | ... | ... | ... | ... | ... | ... | ... | ... | ... |
| | | | " | " | " | " | " | " | " | " | " | " | " | " | " | " | " | " | " | " | " | " | " | " | " | " |
| | | *0108/E+* | ... | ... | ... | ..A | ... | ... | ... | ... | ... | ... | ... | ... | ... | ..C | ... | ... | ... | ... | ... | ... | ... | ..A | ... | ... |
| | | | " | " | " | " | " | " | " | " | " | " | " | " | " | " | " | " | " | " | " | " | " | " | " | " |
| | | *0109/E+* | ... | ... | ... | ..A | ... | ... | ... | ... | ... | ... | ... | ... | ... | ..C | ... | ... | ... | ... | ... | ... | ... | ... | ..A | ... |
| | | | " | " | " | " | " | " | " | " | " | " | " | " | " | " | " | " | " | " | " | " | " | " | " | " |
| | | *0110/E+* | ... | ... | ... | ... | ... | ... | ... | ... | ... | ... | ... | ... | ... | ... | ... | ... | ... | ... | ... | ... | ... | ... | ..A | ... |
| | | | " | " | " | " | " | " | " | " | " | " | " | " | " | " | " | " | " | " | " | " | " | " | " | " |
| | | *0111/E+* | ... | ... | ... | ... | ... | ... | ... | ... | ... | ... | ... | ... | ... | ... | ... | ..T | ... | ... | ... | ..T | ... | ... | ... | ... |
| | | | " | " | " | " | " | " | " | " | " | " | " | " | " | " | " | " | " | " | " | " | " | " | " | " |
| | | *0112/E+* | ... | ..C | ... | ..A | ... | ... | ... | ... | ... | ... | ... | ... | ... | ..C | ... | ..T | ... | ... | ... | ... | ... | ... | ... | ... |
| | | | " | " | " | " | " | " | " | " | " | " | " | " | " | " | " | " | " | " | " | " | " | " | " | " |
| | | *0113/E+* | ... | ..C | ... | ..A | ... | ... | ... | ... | ... | ... | ... | ... | ... | ..C | ... | ..T | ..A | ... | ... | ... | ... | ... | ... | ... |
| | | | " | " | " | " | " | " | " | " | " | " | " | " | " | " | " | " | " | " | " | " | " | " | " | " |
| | | *0114/E+* | ..C | ... | ... | ..A | ... | ... | ... | ... | ... | ... | ... | ... | ... | ... | ... | ... | ... | ... | ... | ... | ... | ... | ... | ... |
| | | | " | " | " | " | " | " | " | " | " | " | " | " | " | " | " | " | " | " | " | " | " | " | " | " |
| | | *0115/E+* | ..C | ... | ... | ... | ... | ... | ... | ... | ... | ..T | ... | ... | ... | ..C | ... | ... | ... | ... | ... | ... | ... | ... | ... | ... |
| | | | " | " | " | " | " | " | " | " | " | " | " | " | " | " | " | " | " | " | " | " | " | " | " | " |
| | *?* | *0001/EU1* | ... | ... | ... | ... | ... | ... | ^CC | ... | ... | ... | ... | ... | ... | ... | ... | ... | ... | ... | ... | ... | ... | ... | ... | ... |
| | | | " | " | " | " | " | " | *FS+2* | " | " | " | " | " | " | " | " | " | " | " | " | " | " | " | " | " |
| | | *0002/EU2* | ... | ... | ... | ..A | ... | ... | ... | ... | ... | ... | A.. | ... | ... | ..C | ... | ... | ... | ... | ... | ... | ... | ... | ..A | ... |
| | | | " | " | " | " | " | " | " | " | " | " | *Met* | " | " | " | " | " | " | " | " | " | " | " | " | " |
| | | *0003/EU3* | ... | ... | ... | ... | ... | ... | ... | ... | ... | ... | ... | ... | ... | ... | ... | ... | ... | ... | ... | ... | ... | ... | A.. | ... |
| | | | " | " | " | " | " | " | " | " | " | " | " | " | " | " | " | " | " | " | " | " | " | " | *Thr* | " |
| | | *0004/EU4* | ... | ..C | ... | ..A | ... | ... | ... | ..T | ... | ... | ... | ... | ... | ..C | ... | ... | ... | ... | ... | ... | ... | ... | ... | ... |
| | | | " | " | " | " | " | " | " | *Leu* | " | " | " | " | " | " | " | " | " | " | " | " | " | " | " | " |
| | *E. Eur Black (Domestic)* | *0201/ED1* | ... | ... | ... | ..A | ... | ... | ... | ... | ... | ... | A.. | .C. | ... | ..C | ... | ... | ... | ... | ... | ... | ... | ... | ..A | ... |
| | | | " | " | " | " | " | " | " | " | " | " | *Met* | *Pro* | " | " | " | " | " | " | " | " | " | " | " | " |
| | | *0202/ED1* | ... | ... | ... | ..A | ... | ... | ... | ... | ... | ... | A.. | .C. | ... | ..C | A.. | ... | ... | ... | ... | ... | ... | ... | ..A | ... |
| | | | " | " | " | " | " | " | " | " | " | " | *Met* | *Pro* | " | " | *Ile* | " | " | " | " | " | " | " | " | " |
| | | *0203/ED1* | ... | ... | ... | ..A | ... | ... | ... | ... | ... | ... | A.. | .C. | ... | ..C | ... | ..T | ... | ... | ... | ... | ... | ... | ..A | ... |
| | | | " | " | " | " | " | " | " | " | " | " | *Met* | *Pro* | " | " | " | " | " | " | " | " | " | " | " | " |
| | | *0204/ED1* | ... | ..C | ... | ..A | ... | ... | ... | ... | ... | ... | A.. | .C. | ... | ..C | A.. | ... | ... | ... | ... | ... | ... | ... | ..A | ... |
| | | | " | " | " | " | " | " | " | " | " | " | *Met* | *Pro* | " | " | *Ile* | " | " | " | " | " | " | " | " | " |
| | *W. Eur Black (Domestic)* | *0301/ED2* | ... | ... | ... | ... | ... | ... | ... | ... | ... | ... | ... | ... | ... | ... | ... | A.. | ... | ... | ... | ... | ... | ... | ... | ... |
| | | | " | " | " | " | " | " | " | " | " | " | " | " | " | " | " | *Asn* | " | " | " | " | " | " | " | " |
| | *Recessive Red (Domestic)* | *0401/e* | ... | ... | ... | ... | ... | ... | ... | ... | ... | ... | ... | ... | ... | ... | ... | ... | ... | .T. | ... | ... | ... | ... | A.. | ... |
| | | | " | " | " | " | " | " | " | " | " | " | " | " | " | " | " | " | " | *Val* | " | " | " | " | *Thr* | " |
| | *Spotted (Domestic)* | *0501/EP* | ... | ... | ... | ... | ... | ... | ^CC | ... | ... | ... | ... | ... | ... | ... | ... | A.. | ... | ... | ... | ... | ... | ... | ... | ... |
| | | | " | " | " | " | " | " | *FS+2* | " | " | " | " | " | " | " | " | *Asn* | " | " | " | " | " | " | " | " |
| | | *0502/EP* | ... | ... | ... | ... | ... | A.. | ^CC | ... | ... | ... | ... | ... | ... | ... | ... | A.. | ... | ... | ... | ... | ... | ... | ... | ... |
| | | | " | " | " | " | " | *Thr* | *FS+2* | " | " | " | " | " | " | " | " | *Asn* | " | " | " | " | " | " | " | " |
| | | *0503/EP* | ... | ... | ... | ... | ... | ... | ^CC | ... | ... | ... | ... | ... | ... | ... | ... | A.. | ... | ... | T.. | ... | ... | ... | ... | ... |
| | | | " | " | " | " | " | " | *FS+2* | " | " | " | " | " | " | " | " | *Asn* | " | " | *Trp* | " | " | " | " | " |
| | *Hawaii's Black Feral Pigs* | *0601/EH* | ... | ... | ... | ..A | ... | ... | ... | ... | ... | ... | ... | ... | ... | ..C | ... | A.. | ... | ... | ... | ... | ... | ..A | ... | ... |
| | | | " | " | " | " | " | " | " | " | " | " | " | " | " | " | " | *Asn* | " | " | " | " | " | " | " | " |

| | | | | | | | | | | | | | | | | | | | | | | | | | | |
|---|---|---|---|---|---|---|---|---|---|---|---|---|---|---|---|---|---|---|---|---|---|---|---|---|---|---|
| *Sus sp. (ISEA)* | *WT S. barbatus (Borneo)* | *0701/EB1* | ...<br>" | ..C<br>" | A..<br>*Ser* | ...<br>" | ..T<br>" | ...<br>" | ...<br>" | ...<br>" | ...<br>" | ...<br>" | ...<br>" | ...<br>" | ...<br>" | ..C<br>" | ...<br>" | ...<br>" | ...<br>" | ...<br>" | ...<br>" | ...<br>" | ...<br>" | ...<br>" | ...<br>" | ...<br>" |
| | | *0702/EB2* | ...<br>" | ..C<br>" | A..<br>*Ser* | ...<br>" | ..T<br>" | ...<br>" | ...<br>" | ...<br>" | ...<br>" | ...<br>" | ...<br>" | ...<br>" | ...<br>" | ..C<br>" | ...<br>" | ...<br>" | ...<br>" | ...<br>" | ...<br>" | ...<br>" | .T.<br>*Val* | ...<br>" | ...<br>" | ...<br>" |
| | *WT S. verrucosus (Java)* | *0801/EJ* | ...<br>" | ..C<br>" | A..<br>*Ser* | ..A<br>" | ..T<br>" | ...<br>" | ...<br>" | ...<br>" | ..T<br>" | ...<br>" | ...<br>" | ...<br>" | ...<br>" | ..C<br>" | ...<br>" | ...<br>" | ...<br>" | ...<br>" | ...<br>" | ...<br>" | ...<br>" | ...<br>" | ...<br>" | ...<br>" |
| | *WT S. celebensis (Sulawesi)* | *0901/ES* | ...<br>" | ..C<br>" | ...<br>" | ...<br>" | ..T<br>" | ...<br>" | ...<br>" | ...<br>" | ...<br>" | ...<br>" | ...<br>" | ...<br>" | ...<br>" | G.C<br>*Asp* | ...<br>" | ...<br>" | ...<br>" | ...<br>" | ...<br>" | ...<br>" | ...<br>" | ...<br>" | ...<br>" | ...<br>" |
| | *WT S. cebifrons (Visayan)* | *1001/EV* | ...<br>" | ..C<br>" | ...<br>" | ...<br>" | ..T<br>" | ...<br>" | ...<br>" | A..<br>*Ile* | ...<br>" | ...<br>" | ...<br>" | ...<br>" | ...<br>" | ..C<br>" | ...<br>" | ...<br>" | ...<br>" | ...<br>" | ...<br>" | ...<br>" | ...<br>" | ...<br>" | ...<br>" | ...<br>" |
