# Supplementary material for: A novel MC1R allele for black coat colour reveals the Polynesian ancestry and hybridization patterns of Hawaiian feral pigs
Source: R Soc Open Sci. 2016 Sep 7;3(9):160304. doi: 10.1098/rsos.160304 (PMC5043315; doi:10.1098/rsos.160304)
Supplement: Supp Figure legends [file rsos160304supp4.docx]

**A Novel *MC1R* allele for black coat colour reveals the Polynesian ancestry and hybridisation patterns of Hawaiian feral pigs**

**Supplementary Figure and Table Legends**

**Supplementary Figure 1.** Median-joining network of porcine *MC1R/E* alleles. Nomenclature, node configuration, and denotation of non-synonymous changes follow Figure 1.

**Supplementary Figure 2.** Alignment for *MC1R/E* nucleotide sequences discussed in this study. An ellipsis (…) and ditto mark (“) indicate that the three base pairs and/or corresponding amino acid at a given position is identical to the 0101 reference sequence. Substitutions that lead to a change in the amino acid are represented by coloured cells. Hawaiian and Polynesian sequences are highlighted in yellow.

**Supplementary Figure 3.** Median-joining network of porcine *MC1R/E* alleles with node size proportionate to the representation of each corresponding haplotype within the dataset. Nomenclature follows Figure 1.

**Supplementary Table 1.** Haplotype and sample acquisition data for Hawaiian pigs considered in this study. Highlighted cells represent novel MC1R haplotypes.

**Supplementary Table 2.** Mitochondrial haplotypes and accession numbers for pulled nucleotide sequences.

**Supplementary Table 3.** Samples in this study contributed by Laurent Frantz. All samples can be found in the European Nucleotide Archive (ENA) under the accession numbers ERP011076 and PRJEB9922.

**Supplementary Table 4.** Samples in this study collated from publically available data.

**Supplementary Table 5.** Frequencies for all *MC1R/E* genotypes considered in this study. Nomenclature follows Table 1.
